# Dataset for small RNA sequencing of murine dendritic cell-derived exosomal miRNAs

**DOI:** 10.1016/j.dib.2026.113277

**Published:** 2026-09-22

**Authors:** Rao Chen, Yikai Wang, Xiaofei Li

**Affiliations:** a Department of Blood Transfusion, Beijing Friendship Hospital, Capital Medical University, Beijing, China; b Department of Sports Medicine, Peking University Third Hospital, Institute of Sports Medicine of Peking University, Beijing, China

**Keywords:** Dendritic cell-derived exosomes, small RNA sequencing, exosomal miRNA, Bone-marrow-derived dendritic cells, Quality control, target enrichment, osteoarthritis

## Abstract

This data article describes a murine dendritic cell-derived exosomes (DEXs) small RNA sequencing dataset generated to support reuse of exosomal miRNA profiles in studies of immune regulation and osteoarthritis-related mechanisms. Data collection began with BMDC isolation, culture and characterization via flow cytometry; DEXs were purified from imDC supernatant using a CP-100WX Ultracentrifuge (HITACHI, Japan) with a standardized ultracentrifugation protocol. The dataset contains six paired-end FASTQ files from three small RNA sequencing libraries, DEXs1, DEXs2 and DEXs3, prepared from the DEX RNA material documented in the project files. Three downstream small RNA libraries were then constructed and sequenced on an Illumina PE150 platform. The processed dataset includes sequencing-quality tables, Rfam-filtering and genome-mapping summaries, miRNA count and TPM-normalized expression matrices, shared-miRNA tables, target-gene tables for the top shared miRNAs, and GO, KEGG and Reactome enrichment outputs for predicted and experimentally validated target records. Quality control included FastQC quality assessment, fastp-based read cleaning, removal of adapter-contaminated reads, reads with >10% unknown bases, and reads with >50% low-quality bases, followed by reporting of Q20, Q30, GC content and clean-read metrics. The Zenodo record contains the raw FASTQ.gz files, processed table archive, figure archive, QC report, MD5 checksum file and R workflow script, supporting reuse and reproducible review of the dataset.

Specifications Table

**Table:** 

Subject	Health Sciences; Medical Sciences; Molecular Biology; Immunology
Specific subject area	Small RNA/miRNA sequencing data from murine dendritic cell-derived exosomes.
Type of data	Raw paired-end FASTQ folders; MD5 checksum text file; sequencing and library QC report; processed table folder; PNG figure folder; R analysis script.
Data collection	DEX RNA was extracted from a documented murine exosome sample, inspected by Quantus Fluorometer and Qsep100, converted into three QIAseq small RNA libraries, and sequenced using an Illumina PE150 workflow. Read 1 was used for small RNA analysis in the supplier report.
Data source location	Experimental work and dataset curation were associated with Peking University Third Hospital, Beijing, China, and collaborating authors' institutions listed in the Article Information section.
Data accessibility	Repository name: Zenodo Data identification number: 10.5281/zenodo.21231110 Direct URL to data: https://doi.org/10.5281/zenodo.21231110 Dataset citation: Wang, Y. (2026). Dataset for small RNA sequencing of murine dendritic cell-derived exosomal miRNAs [Data set]. Zenodo. https://doi.org/10.5281/zenodo.21231110.
Related research article	Rao Chen, Yikai Wang, Dendritic cells-derived exosomes induced Tregs generation and C3 degradation to prevent osteoarthritis progression, Materials and Design. Publicated.

## 1 Value of the Data

• The dataset provides raw and processed small RNA sequencing files for DEX-derived exosomal miRNAs, together with explicit sequencing-quality, library-quality and post-filtering metrics.
• Researchers can reuse the count matrix, TPM matrix and shared-miRNA tables to compare DEX miRNA profiles with other exosome or dendritic-cell datasets without repeating the initial sequencing workflow.
• The target-gene and enrichment tables provide reusable predicted and experimentally supported target annotations for the top shared DEX miRNAs.
• The included R workflow script documents data inventory, quality control, cleaning, normalization, sample-level visualization, target-table summarization and figure generation for reproducible review.
• DEXs1, DEXs2, and DEXs3 are three biological replicates derived from three independently cultured BMDC preparations, enabling secondary users to assess biological variability across the DEX miRNA profiles.

## 2 Background

DEXs contain regulatory small RNAs that can be used to study exosome-mediated immune communication. The present dataset was compiled as a transparent small RNA sequencing resource for murine DEX-derived miRNAs. It adds value to the related research article [1] by providing raw sequencing files, processed expression and annotation tables, target-gene records, enrichment outputs, quality-control documentation and an executable R workflow. The data article focuses on dataset generation, curation, quality control and reuse; biological interpretation of DEX function is reserved for the associated research article.

## 3 Data Description

The public Zenodo record is organized as individual raw FASTQ.gz files and supporting archives/files so that the raw sequencing data, integrity checks, QC report, processed tables, figures and analysis script can be located directly [2]. Table 1 lists the final Zenodo file structure used for the data article.

**Table 1:** Zenodo repository file list for the DEX miRNA sequencing dataset.

Zenodo file	Size	Description
01.Tables.zip	4.9 MB	Compressed archive of processed CSV tables, including sequencing QC summaries, Rfam and mapping summaries, miRNA count/TPM matrices, shared-miRNA records, target-gene tables and enrichment outputs.
03.PNG_Figures.zip	1.8 MB	Compressed archive of grouped PNG figure panels summarizing sequencing QC, sample relationships, miRNA expression landscape and target-gene enrichment outputs.
260,531,568-mouse-1_S76_L002_R1_001.fastq.gz	758.8 MB	Raw read 1 FASTQ.gz file for the DEXs1 small RNA library.
260,531,568-mouse-1_S76_L002_R2_001.fastq.gz	786.1 MB	Raw read 2 FASTQ.gz file for the DEXs1 small RNA library.
260,531,568-mouse-2_S77_L002_R1_001.fastq.gz	571.1 MB	Raw read 1 FASTQ.gz file for the DEXs2 small RNA library.
260,531,568-mouse-2_S77_L002_R2_001.fastq.gz	529.9 MB	Raw read 2 FASTQ.gz file for the DEXs2 small RNA library.
260,531,568-mouse-3_S0_L000_R1_000.fastq.gz	723.1 MB	Raw read 1 FASTQ.gz file for the DEXs3 small RNA library.
260,531,568-mouse-3_S0_L000_R2_000.fastq.gz	710.9 MB	Raw read 2 FASTQ.gz file for the DEXs3 small RNA library.
260,531,568_Sequencing_and_Library_QC_Report.docx	74.4 kB	Combined report containing raw/clean read QC, Q20/Q30/GC metrics, RNA and library-quality information, library size and run-risk notes.
DEXs_miRNA_full_reproducible_analysis.R	24.7 kB	R workflow for raw-data inventory, checksum recording, QC table parsing, normalization, visualization and downstream table/figure generation.
MD5.txt	628 Bytes	Checksum file for verifying the integrity of the deposited raw FASTQ files.

The six FASTQ.gz files contain the raw sequencing component. DEXs1 and DEXs2 were sequenced on 2026–06–08 and DEXs3 was sequenced on 2026–06–10 after additional sequencing. The sample name, library name, platform ID, and library type were shown in Table 2. Each sample is represented by R1 and R2 FASTQ.gz files, and MD5.txt provides checksum information for raw-file integrity verification. The supplier report states that read 1 was used for small RNA analysis.

**Table 2:** Sample, library and biological-representativeness metadata extracted from the project files.

Sample	Library name	Platform ID	Library type
DEXs1	260,531,568-mouse-1	JB26NL50534_a	Small RNA library
DEXs2	260,531,568-mouse-2	JB26NL50535_a	Small RNA library
DEXs3	260,531,568-mouse-3	JB26NL50536_a	Small RNA library

DEXs1, DEXs2, and DEXs3 were generated from three independently cultured BMDC preparations and therefore represent three biological replicates. A separate small RNA sequencing library was constructed for each biological replicate, supporting assessment of biological variability and sample-level quality.

The raw and clean read metrics in Table 3 are accompanied by the grouped QC and sample-relationship panels in Fig. 1, which show per-sequence quality distributions, hierarchical sample clustering, PCA and a normalized sample-distance heatmap Table 4.

**Table 3:** Sequencing and post-cleaning quality summary.

Sample	Raw reads per mate	Clean reads per mate	Raw Q20 range	Raw Q30 range	Clean Q20 range	Clean Q30 range	Clean GC
DEXs1	14,037,583	13,909,807	93.782–94.015%	88.062–88.382%	99.024–99.043%	96.614–96.845%	50%
DEXs2	10,070,314	9982,992	91.888–94.624%	85.291–89.801%	98.980–99.164%	96.649–97.047%	50%
DEXs3	13,243,514	13,134,498	93.743–94.799%	87.913–89.880%	99.072–99.240%	96.857–97.198%	50%

**Fig. 1:**
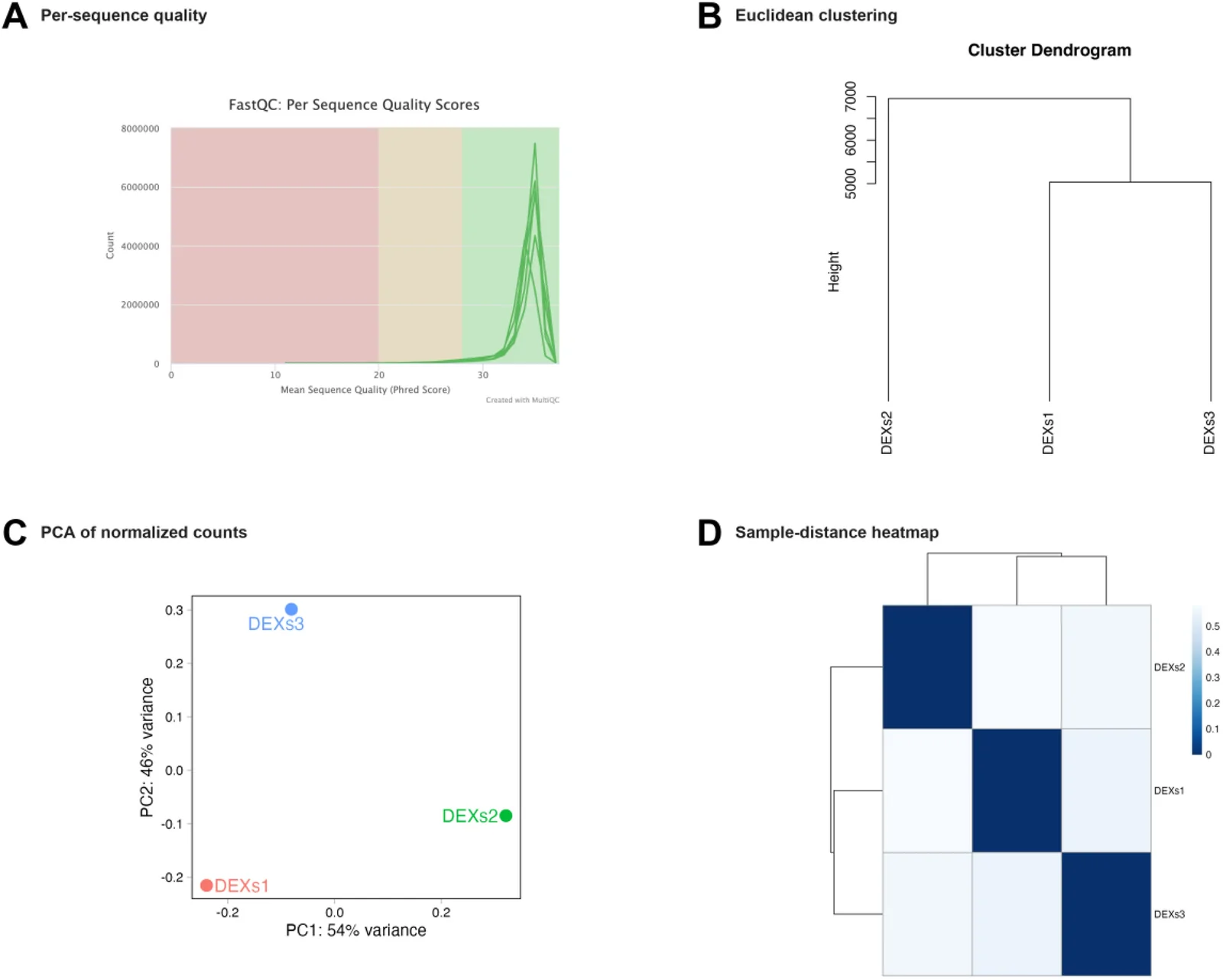
Sequencing quality and sample-level relationships. (A) FastQC per-sequence quality scores. (B) Euclidean clustering of DEXs1-DEXs3. (C) PCA of normalized miRNA counts. (D) DESeq2-normalized sample-distance heatmap.

**Table 4:** Processed report tables included in the data package.

Report table	Rows	Main data type	Use in dataset
Table 2	6	Raw/clean read QC	Q20, Q30, GC and read counts for R1/R2
Table 3	28	Small RNA length counts	Length distribution for DEXs1-DEXs3
Table 4	3	Rfam categories	ncRNA annotation counts
Table 5	3	Mapping rate	Rfam-filtered read mapping rate
Table 6	585	miRNA expression	Counts and normalized expression values
Table 7	324	Shared miRNAs	miRNAs detected in all three libraries
Table 8	58,086	Predicted targets	Predicted target records
Table 9	39,506	Validated targets	Experimentally supported targets
Table 10	17,724	Predicted-target GO	GO enrichment
Table 11	361	Predicted-target KEGG	KEGG enrichment
Table 12	1616	Predicted-target Reactome	Reactome enrichment
Table 13	20,341	Validated-target GO	GO enrichment
Table 14	364	Validated-target KEGG	KEGG enrichment
Table 15	1691	Validated-target Reactome	Reactome enrichment
Table 16	23,348	Target overlap	Predicted/validated overlap

The processed miRNA tables in 01.Tables.zip are summarized visually in Fig. 2, including small RNA length distribution, overlap of detected miRNAs among the three libraries and the top shared miRNAs by total read count. Target-gene and enrichment outputs are visualized in Fig. 3 for predicted targets and Fig. 4 for experimentally validated targets, and the source PNG panels are deposited in 03.PNG_Figures.zip.

**Fig. 2:**
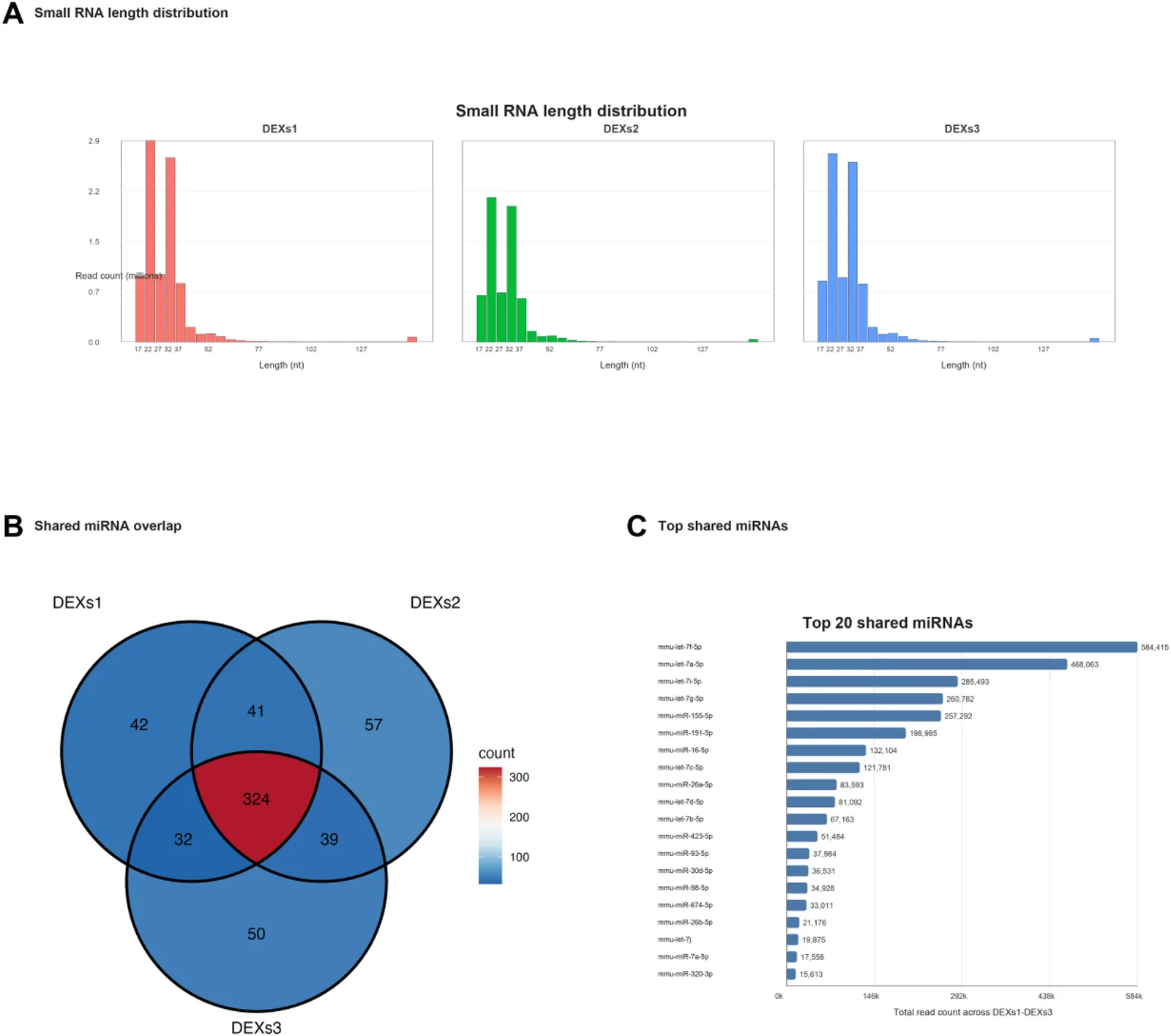
Small RNA and miRNA expression landscape. (A) Small RNA length distribution. (B) miRNA overlap among DEXs1, DEXs2 and DEXs3. (C) Top 20 shared miRNAs ranked by total read count across the three libraries.

**Fig. 3:**
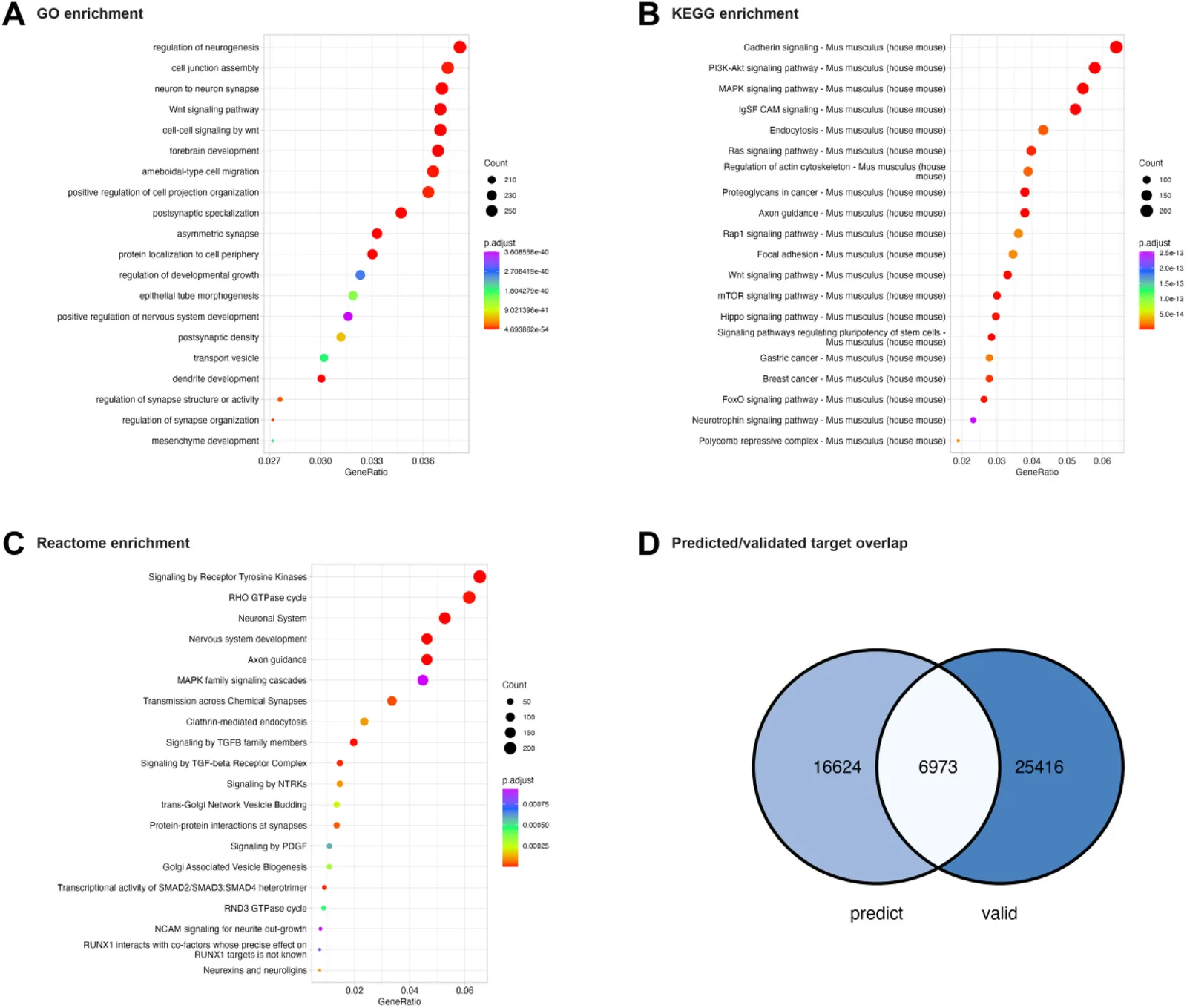
Predicted target-gene enrichment for the top shared miRNAs. (A) GO enrichment. (B) KEGG pathway enrichment. (C) Reactome pathway enrichment. (D) Overlap between predicted and validated target records.

**Fig. 4:**
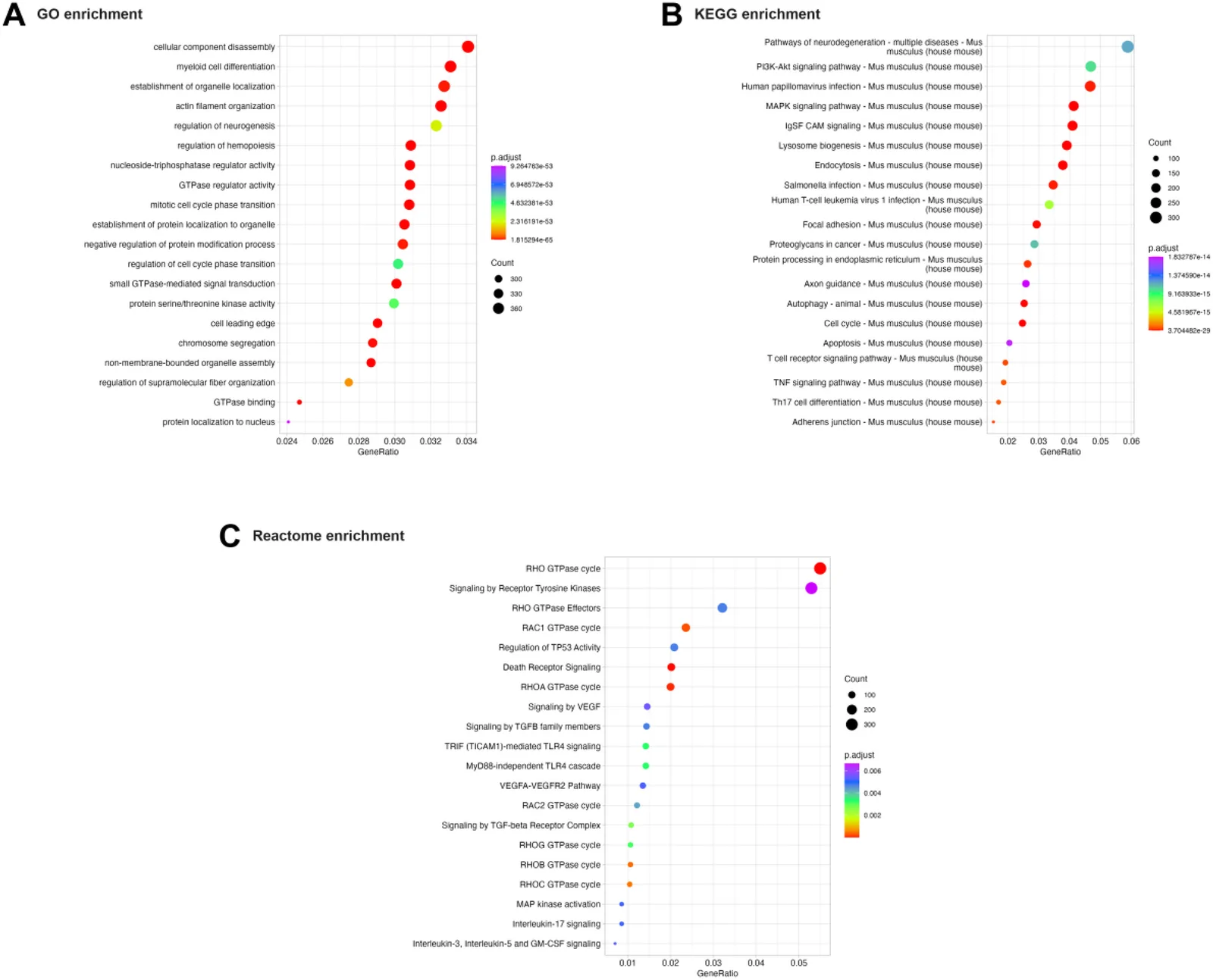
Experimentally validated target-gene enrichment for the top shared miRNAs. (A) GO enrichment. (B) KEGG pathway enrichment. (C) Reactome pathway enrichment.

## 4 Experimental Design, Materials and Methods

### 4.1 Biological source and representativeness

BMDCs were obtained from 6-week-old BALB/c mice according to the related experimental protocol. Bone marrow was flushed, passed through a 70-µm cell strainer, treated with erythrocyte lysis buffer and cultured in RPMI 1640 medium supplemented with 10% exosome-depleted fetal bovine serum (FBS; catalog no iCell-0650), penicillin, streptomycin, l-glutamine, non-essential amino acids, sodium pyruvate, HEPES, 2-mercaptoethanol, GM-CSF and IL-4. Non-stimulated cells after culture were used as immature dendritic cells for DEXs production. Flow-cytometric characterization showed that the CD11c-positive proportion of the imDC population was 83.07 ± 9.17% (mean ± SD, n = 3 independent BMDC preparations). At harvest, defined as the time of conditioned-medium collection for DEX isolation, imDC viability was 94.63 ± 1.47% (mean ± SD, n = 3), as determined by flow-cytometric live/dead staining. Representative CD11c and live/dead staining gating plots from one independent BMDC preparation are shown in Fig. 5. These values document the CD11c-positive proportion of the parental imDC population and its viability at harvest.

**Fig. 5:**
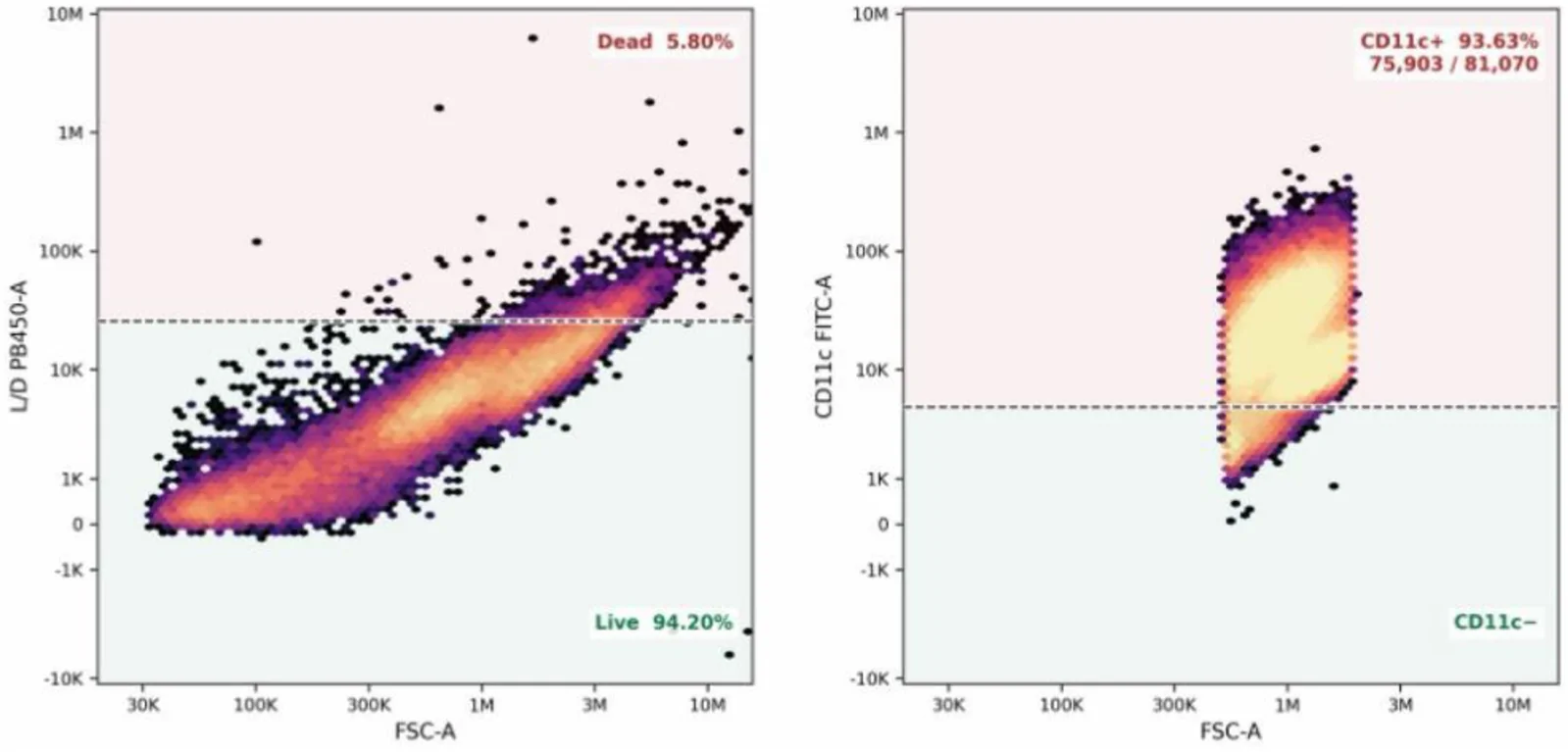
Flow-cytometry gating for viability and CD11c expression. Left, live/dead staining analysis. Right, CD11c staining after the Lymphocytes and Single Cells gates. Percentages are calculated relative to the indicated parent population.

DEXs1, DEXs2, and DEXs3 were obtained from three independently cultured BMDC preparations and processed as three biological replicates. RNA was extracted separately from each DEX preparation, and one small RNA sequencing library was constructed for each biological replicate.

### 4.2 DEXs isolation and RNA quality assessment

DEXs were isolated from immature dendritic-cell culture supernatant by differential centrifugation and ultracentrifugation. Supernatant was centrifuged at 300 x g for 10 min to remove intact cells, 2000 x g for 10 min to remove dead cells, and 10,000 x g for 30 min to remove debris. The remaining supernatant was ultracentrifuged at 100,000 x g for 70 min, washed in PBS and ultracentrifuged again at 100,000 x g for 70 min. The pellet was resuspended in pre-cooled 0.22-um filtered PBS.

DEXs were characterized using transmission electron microscopy (TEM), nanoparticle tracking analysis (NTA), and Western blotting. TEM using a JEM-1400 microscope (JEOL, Japan) demonstrated the characteristic membrane-bound vesicular morphology. NTA using a Particle Metrix ZetaView system (software v8.05.16 SP3; EV_488 SOP) showed a median particle diameter of 145.8 nm (X10-X90, 83.5–252.9 nm; mean, 165.0 nm) and an estimated original-sample concentration of 1.1 × 10^11 particles/mL. Western blotting showed the EV-associated markers CD81, CD63, and TSG101; cytochrome C was not detected in DEXs but was detected in the parental-cell lysate. The corresponding characterization data are provided in Fig. 1A-C of the associated primary research article [1].

RNA was extracted using the miRNeasy Mini Kit (Qiagen, 217,004). RNA concentration was measured using a Quantus Fluorometer (Promega), and RNA size distribution was inspected using a Qsep100 instrument (BIOptic). The RNA/Qsep report recorded an RNA concentration of 2.5 ng/uL and total RNA amount of 62.5 ng.

### 4.3 Small RNA library construction and sequencing

Small RNA libraries were constructed using the QIAseq miRNA Library Kit (Qiagen, 331,505). The workflow included 3′ adaptor ligation, 5′ adaptor ligation, reverse transcription, cDNA purification, PCR amplification and bead-based library size selection. The recorded adaptor sequence was AACTGTAGGCACCATCAATNNNNNNNNNNNNAGATCGGAAGAGCACACGTCTGAACTCCAGTCAC. Library concentration was assessed by Qubit and qPCR, and library size was inspected by Fragment Analyzer. The three libraries had sizes of 187–191 bp and qPCR concentrations of 24.53–28.63 nM. All three were classified as risk-on sequencing because of small insert fragments and, for two libraries, complex peak shapes.

Paired-end sequencing was performed using an Illumina PE150 strategy. DEXs1 and DEXs2 were sequenced on 2026-06-08; DEXs3 was sequenced on 2026-06-10 as an additional sequencing run. Raw output was 4.21 Gb for DEXs1, 3.02 Gb for DEXs2 and 3.97 Gb for DEXs3 in the sequencing QC report.

### 4.4 Quality control and cleaning

The quality-control component referenced in the manuscript corresponds to 260,531, 568_Sequencing_and_Library_QC_Report.docx and to the processed QC tables in 01.Tables.zip. FastQC was used to inspect per-sequence quality-score distributions, which are summarized in Fig. 1A. fastp [6] was used for N-base trimming at read ends, Q20-based filtering and adaptor removal. Reads were removed if they contained adaptor contamination, contained >10% N bases, or had >50% bases with quality score lower than 5. Q20, Q30 and GC content were calculated for raw and clean reads. After cleaning, all six read files showed Q20 values of 98.980–99.240%, Q30 values of 96.614–97.198% and clean GC content of 50%. Sample-level relationships from normalized miRNA counts are shown in Fig. 1B-D.

### 4.5 Mapping, miRNA quantification and downstream tables

The supplier report states that read 1 was used for small RNA analysis. Clean reads were mapped with bowtie [3] to the Rfam database to mark and remove rRNA, tRNA and other ncRNA reads, followed by reference-genome mapping-rate assessment. The analysis report lists Ensembl release 102 and miRBase release 22 [8] as reference resources; because the dataset is murine and the target/enrichment outputs use *Mus musculus* identifiers, the deposited R script keeps reference paths configurable for remapping with user-supplied mouse reference files. miRNA expression was quantified using miRDeep2 [4]; miREvo represents another widely-adopted computational frame work for small-RNA-derived miRNA profiling [5]. Expression values were normalized to transcripts per million using TPM = read count x 1000,000 / total miRNA read count in each sample. The report includes 585 detected miRNA records and 324 miRNAs shared among DEXs1, DEXs2 and DEXs3. The most abundant shared miRNAs in the processed table include mmu-let-7f-5p, mmu-let-7a-5p, mmu-let-7i-5p, mmu-let-7 *g*-5p and mmu-miR-155-5p; these distribution and shared-miRNA summaries are shown in Fig. 2.

DESeq2-normalized expression values were used for PCA and sample-distance visualization [7]. Formal differential expression testing is not presented as a primary analysis for this one-condition dataset; the uploaded R script is configured to skip differential testing unless users supply a valid multi-group sample sheet with sufficient biological replication.

For the top shared miRNAs, predicted target-gene tables and experimentally validated target-gene tables were generated [[9], [10]], followed by GO, KEGG and Reactome enrichment analysis [[11], [12], [13]]. Enrichment tables report GeneRatio, BgRatio, RichFactor, FoldEnrichment, zScore, p-value and Benjamini-Hochberg adjusted p-value where applicable. The data package contains 58,086 predicted target records, 39,506 validated target records and 23,348 overlap target records. The predicted-target enrichment outputs and predicted/validated target overlap are shown in Fig. 3, and the validated-target enrichment outputs are shown in Fig. 4.

### 4.6 Reproducible analysis script

The deposited script DEXs_miRNA_full_reproducible_analysis.R records the workflow from raw FASTQ inventory and MD5 checksums to FASTQ cleaning, QC summaries, optional external mapping, miRNA TPM normalization, PCA, sample-distance heatmap, shared-miRNA extraction, target-table summaries and enrichment-table export. The script does not install software automatically; users should provide local paths to bowtie/Rfam/genome/miRBase resources before enabling remapping. This addresses the reproducibility requirement by making the analysis logic, sample sheet, QC thresholds and output-file structure explicit.

## Limitations

A key limitation of this study is the absence of comparative genomic or small RNA sequencing analyses between dendritic cell-derived exosomes (DEXs) and other exosome subtypes. This restricts our understanding of the unique molecular signatures of DEXs relative to other exosomal populations. Future studies should supplement such comparative analyses to clarify the specific regulatory characteristics of DEXs.

## Ethics Statement

All animal care and experiments were conducted under approval of the Institutional Authority for Laboratory Animal Care of Peking University (approval number: LA2022542).

## Credit Author Statement

**Rao Chen and Yikai Wang:** contributed equally. **Rao Chen:** Writing - original draft, Visualization, Investigation, Formal analysis. **Yikai Wang:** Writing - original draft, Visualization. **Xiaofei Li:** Conceptualization, Experimental design, Funding acquisition, Supervision.
